# Titanium-Tethered Vancomycin Prevents Resistance to Rifampicin in *Staphylococcus aureus in vitro*

**DOI:** 10.1371/journal.pone.0052883

**Published:** 2012-12-20

**Authors:** Martin Rottman, Joel Goldberg, S. Adam Hacking

**Affiliations:** 1 Laboratory for Musculoskeletal Research and Innovation, Department of Orthopaedics, Harvard Medical School and Massachusetts General Hospital, Boston, Massachusetts, United States of America; 2 The Wyss Institute at Harvard Medical School, Boston, Massachusetts, United States of America; 3 EA 3647 Physiopathologie et Diagnostic des Infections Microbiennes, Université Versailles St Quentin, and Laboratoire de Microbiologie, Hôpital Raymond Poincaré, AP-HP, Garches, France; Charité-University Medicine Berlin, Germany

## Abstract

Rifampicin is currently recognized as the most potent drug against Gram positive implant related infections. The use of rifampicin is limited by the emergence of bacterial resistance, which is often managed by coadministration of a second antibiotic. The purpose of this study was to determine the effectiveness of soluble rifampicin in combination with vancomycin tethered to titanium metal as a means to control bacterial growth and resistance *in vitro*. Bacterial growth was inhibited when the vancomycin-tethered titanium discs were treated with *Staphylococcus aureus* inocula of ≤2×10^6^ CFU, however inocula greater than 2×10^6^ CFU/disc adhered and survived. The combination of surface-tethered vancomycin with soluble rifampicin enhanced the inhibitory effect of rifampicin for an inoculum of 10^6^ CFU/cm^2^ by one dilution (combination MIC of 0.008 mg/L versus 0.015 mg/L for rifampicin alone). Moreover, surface tethered vancomycin prevented the emergence of a rifampicin resistant population in an inoculum of 2×10^8^ CFU.

## Introduction

Infection is a persistent complication with a number of implanted devices. In total joint replacement (TJR), implant related infections are debilitating, costly and difficult to treat [1]. Patients with infected implants require prolonged hospitalization and consume a disproportionate amount of healthcare resources [1]. Improved operative techniques and short-term administration of peri-operative antibiotics have proven successful in reducing the overall incidence of prosthetic joint infection (PJI) (1.6% post-op up to 2 years and 0.5% thereafter) [2]. However, PJIs persist and are notoriously difficult to treat with systemic administration of antibiotics.

The management of infected implants often necessitates a highly invasive treatment plan [3], [4] such as the two stage revision procedure [5]. The first stage removes the infected implant and surrounding tissue which is followed by six to twelve weeks of antibiotic treatment. After antibiotic treatment, a second procedure is performed to reconstruct the joint with new implants [6]. A two stage procedure can be a long and costly process. One strategy to potentially improve patient outcome and reduce the burden of care, is a single stage revision [5], [7]. In a single stage procedure, the infected implant is removed, the wound is debrided and cleaned and a new prosthesis is inserted, all during the same surgical setting. Such single stage procedures were initially performed with antibiotic-releasing bone cement but are now also performed using cementless implants. Single stage procedures have been shown to reduce the cost of PJI revision [8], however concerns about the efficacy of treatment and the potential for the recurrence of infection persist.

For TJRs performed without cement, titanium alloys are often the preferred implant materials because of their biocompatibility and elastic modulus [9]. The inherent biocompatibility of titanium is attributed to a heterogeneous layer rich in oxygen that spontaneously forms on the titanium surface. Strategies have been developed to tether various molecules of biological interest such as antibiotics to the titanium surface. Antibiotics such as ampicillin [10], daptomycin [11] and vancomycin [12]–[16] maintain bioactivity when covalently linked (tethered) to a solid surface. Several techniques have been described for the covalent linkage to a titanium oxide surface, where the organic-metal connection is made through silane [17], phosphate [18], phosphonate [19] or catechol [20] linkers. One strategy involving vancomycin is the covalent bonding of the antibiotic to a titanium alloy surface through a short polyethylene (PEG) tether using a silane linker [14].

Vancomycin inhibits cell wall biosynthesis by binding to terminal *D*-alanyl-*D*-alanine residues of NAG/NAM peptides, preventing their cross-linking [21]. Surface bound vancomycin can prevent biofilm formation on titanium surfaces *in vitro*, and *in vivo* [22]. The tethering of vancomycin to the titanium surface is an advantageous method of antibiotic prophylaxis since it precludes exposure of the recipient's flora to antimicrobial agents and the need for intravenous delivery. Since vancomycin is active against 80% of microorganisms causing PJIs [23] it is also a relevant choice for tethering to implant surfaces for the treatment of infection.

The coadministration of vancomycin and rifampicin is the mainstay in the treatment of methicillin-resistant *Staphylococcus aureus* implant infection [24] and this combination is often used for treatment durations exceeding six to twelve weeks. Rifampicin is a drug of choice since it has excellent bioavailability, tissue diffusion, can be administered orally once daily, and remains active on biofilms [25]. Rifampicin is commonly coadministered with other antibiotics since the emergence of resistant bacteria is a common problem [26]. While vancomycin is an effective antibiotic for the treatment of methicillin-resistant *Staphylococcus aureus*, it has a poor tissue diffusion profile and can only be administered intravenously with required serum level monitoring. To ensure adequate bioavailability, administration of vancomycin often precedes rifampicin and delays the beneficial effects of rifampicin therapy.

To overcome these issues, the combination of implant-tethered vancomycin with an oral regimen of rifampicin may be a practical method to achieve an optimal antimicrobial environment immediately following the implantation of a cementless implant for revision surgery. In a clinical setting, intravenous vancomycin could be coadministered to guard against resistance from rifampicin monotherapy outside the joint space. The purpose of this study was to determine if the combination of surface-tethered vancomycin and soluble rifampicin would prevent the emergence of resistance to rifampicin in *S. aureus in vitro.*

## Materials and Methods

### Bacterial strain and culture

All microbial studies were conducted using *Staphylococcus aureus* ATCC strain 25923. Lyophilized stock was subcultured on Tryptic Soy agar plates with 5% sheep blood (BD Diagnostics, Sparks, MD) and a 10 ml preculture performed in Tryptic Soy Broth (TSB) (DIFCO, BD Diagnostics). TSB (1 L) was seeded with the preculture and grown to an optical density of 0.5 at 600 nm as measured on a Nanodrop 2000c spectrophotometer (Thermo) and then cooled on ice. The culture was pelleted and the pellet washed with PBS (pH 7.2). Following a second centrifugation the pellet was resuspended in PBS with 10% glycerol and 1 ml aliquots frozen and stored at –80°C. Thawed aliquots were titrated by plating serial dilutions on agar medium.

### Preparation of Titanium Discs

Grade 5-ELI titanium alloy (TiAl6V4) discs (21 mm diameter ×1 mm thickness) were fabricated in-house from rod stock. All other materials were purchased from Sigma Aldrich (St. Louis, MO) unless otherwise noted. Using standard metallurgical techniques, the titanium discs were polished until they had a mirror finish (S_a_∼0.02 µm). After polishing, discs were cleaned for 30 minutes at 80°C in a 2% solution of Liquinox (White Plains, NY) in an ultrasonic bath (Crest Ultrasonics, Tenton NJ). Following cleaning, discs were passivated by treatment in 50% nitric acid at 50°C with sonication for 1 h and then washed with deionized H_2_O and dried in a 100°C oven. Discs were placed in self-sealing sterilization pouches (Defend, Hauppauge NY) and steam sterilized at 135°C for 20 minutes. At this point the sterilized discs were split into 2 groups, control and experimental.

### Tethering of vancomycin to titanium discs

The experimental titanium discs were identical to control discs in every respect except that vancomycin was tethered to the disc surface following the procedure of Jose et al. [14]. Briefly, the tethering of vancomycin to the titanium surface was achieved as follows: experimental discs were submerged in a sealed glass chamber containing a solution of aminopropyltriethoxysilane (1.2 ml, 5 mmol) in anhydrous toluene (50 ml) and heated to 120°C for 3 h. The discs were cooled, washed sequentially with ethyl acetate, ethanol and deionized H_2_O, and dried under vacuum. A short polyethylene glycol (PEG) based tether was then linked to the exposed amino groups by treating the discs with 2-[2-(fmoc-amino)ethoxy]ethoxyacetic acid (0.3 g, 0.8 mmol), COMU (0.34 g, 0.8 mmol) and *i*-Pr_2_NEt (0.3 ml, 1.6 mmol) in dimethylformamide (40 ml) for 1 h at 22°C. After washing (dimethylformamide), the fmoc group was removed by treatment with 25% piperidine in dimethylformamide (50 ml), and the discs were washed again (dimethylformamide and 2-propanol). The 2-[2-(fmoc-amino)ethoxy]ethoxyacetic acid coupling and deprotection protocol was repeated to double the length of the PEG linker. The antibiotic was then covalently linked by treating the discs with vancomycin hydrochloride (Hospira, Inc., Lake Forest, Il) (0.4 g, 0.27 mmol), COMU (0.12 g, 0.3 mmol) and *i*-Pr_2_NEt (0.4 ml, 2.2 mmol) in dimethylformamide (50 ml) for 16 h at 22°C. The antibiotic-coupled discs were then washed (sequentially with dimethylformamide, 2-propanol and deionized H_2_O) and dried and stored under vacuum at ambient temperature until ready for use.

### Competitive Fluorescent Linked ImmunoSorbent Assay (FLISA)

Control and experimental discs were placed in 12-well plates (BD Falcon), blocked with 1% BSA in PBS (3 h at 37°C) and washed with 0.05% PBS-Tween 20 (3×15 min under orbital agitation). Solutions of competing soluble vancomycin (0.25 ml, 0–25 mg/well) were added to each well followed by 0.25 ml of 1∶5,000 polyclonal rabbit anti-vancomycin antibody (Pierce Thermo). After 45 min at 37°C, each well was washed (3×0.05% PBS-Tween 20), treated with 0.5 ml 1∶5000 Alexa 488-labeled goat anti-rabbit antibody (Invitrogen) and incubated in the dark at 37°C for 45 min. The discs were washed (3×0.05% PBS-Tween 20), and transferred to new plates with PBS (1 ml) in each well. Fluorescence (excitation 488 nm, emission 525 nm) was read on a Biotek H1 synergy plate reader (Biotek, Winooski, VT, USA). All assays were performed in triplicate.

### Resazurin biofilm assay and measurement of surface antimicrobial activity

Control and experimental discs were placed in 12-well plates, containing Mueller Hinton broth (MHB) (1 ml) and seeded with ten-fold serial dilutions of thawed stock bacteria (2×10^6^ to 2×10^3^ CFU/well). The plates were centrifuged (10 min at 1000 G) and incubated at 36°C for 18 h. The discs were washed gently to remove unattached bacteria (3×PBS) and transferred to a fresh plate. Fresh MHB supplemented with 200 µM resazurin (Sigma) (resazurin MHB) was added and incubated for 1 h. The fluorescence of 100 µl aliquots of the resazurin MHB was then measured (excitation 560 nm, emission 594 nm) to assess the reduction of resazurin into the fluorescent resorufin compound, a measure of metabolic activity of the disc-associated biofilm [27], [28]. All assays were performed in triplicate.

### Synergy assay

A classic checkerboard synergy assay was performed according to CLSI recommendations in resazurin MHB with an inoculum of 5×10^4^ CFU/well under a final volume of 200 µl. The synergy between vancomycin tethered to titanium and soluble rifampicin was evaluated by inoculating control and experimental discs with 5×10^6^ CFU/well, then incubating with two-fold serial dilutions of rifampicin in resazurin MHB (2 ml/well). The synergy was determined by comparing the lowest rifampicin concentration capable of inhibiting growth on vancomycin treated surface to the lowest concentration capable of inhibiting growth on a control titanium surface. Inhibition of growth was determined by colorimetric evaluation of the lack of resazurin reduction.

### Confocal laser scanning microscopy of control and experimental discs

Bacterial inocula suspended in MHB were added to control or experimental discs placed in 12 well plates and centrifuged 10 minutes at 1000 G. Discs were gently washed three times in PBS and stained with a live/dead BacLight bacterial viability kit (Invitrogen) according to the manufacturer's recommendations, fixed in 4% paraformaldehyde containing PBS and imaged using an upright Zeiss LSM 710 confocal microscope. Live bacteria were stained with Syto9 (green) and dead bacteria stained with both Syto9 and propidium iodide (yellow).

### Statistical analysis

The significance of quantitative data was analyzed using Student's t test and a p value <0.05 was considered to be significant.

## Results

### Vancomycin tethered to the titanium surface can be quantified by a competitive Fluorescent-Linked ImmunoSorbent Assay

**Figure 1:**
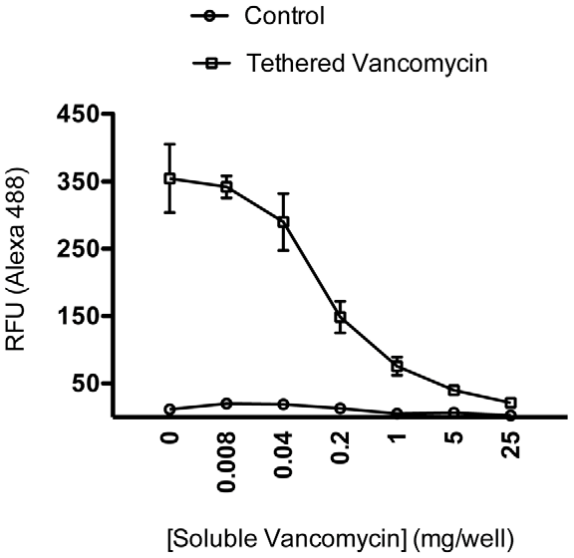
Quantification of vancomycin covalently tethered to the titanium surface. The amount of vancomycin tethered to the titanium surface was determined using a competitive Fluorescent-Linked ImmunoSorbent Assay. Anti-vancomycin antibody was mixed with 5-fold dilutions of soluble vancomycin and then incubated with the surface-tethered vancomycin. After washing and detecting with a fluorescent secondary antibody, the amount of vancomycin bound to the disc surface was calculated to be 0.20 mg [+/−0.06] (average from a duplicate assay of 3 batches [+/− standard deviation]) or 0.14 nmol. This corresponds to 8.3×10^13^ vancomycin molecules per disc face, or one vancomycin molecule per 3.8 nm^2^.

The amount of vancomycin tethered to the titanium surface was determined using a competitive Fluorescent Linked ImmunoSorbent Assay. (Figure 1) Anti-vancomycin antibody was mixed with varying amounts of soluble drug and then allowed to equilibrate with the surface-tethered vancomycin. After washing and specifically detecting vancomycin with a fluorescent secondary antibody, the amount of vancomycin tethered to the disc surface was established by determining the concentration of soluble competitor that reduced the measured fluorescence by 50%. From this result it was determined that 0.20 mg ±0.06 of tethered vancomycin (average from a duplicate assay of 3 batches) was attached per disk face which also corresponded to 0.14 nmol or 8.3×10^13^ vancomycin molecules per disc face or one vancomycin molecule per 3.8 nm^2^.

### The antimicrobial effect of vancomycin tethered to titanium was inoculum dependent

**Figure 2:**
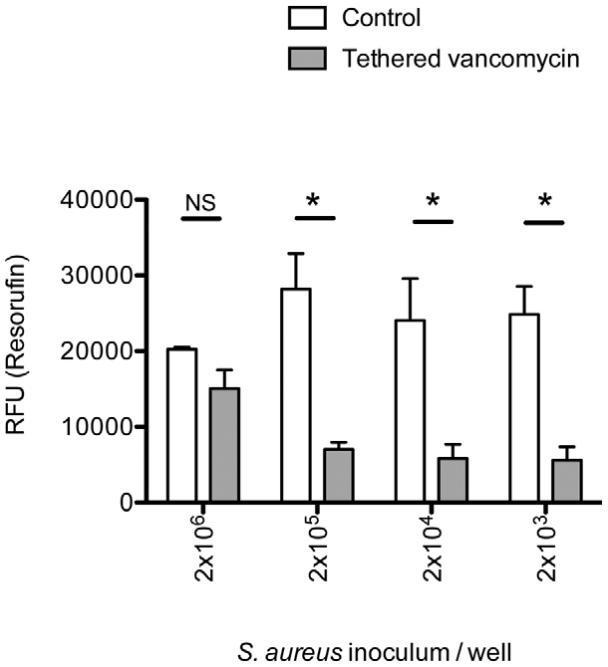
The antimicrobial effect of vancomycin tethered titanium was inoculum dependent. Vancomycin tethered or control discs were seeded with ten-fold dilutions of *S. aureus* stock suspension and incubated overnight. The metabolically active bacteria adhering to the disc surface were quantified by measuring resorufin fluorescence. Vancomycin-tethered discs significantly inhibited the adhesion and survival of 2×10^3^, 2×10^4^ and 2×10^5^ CFU compared to control titanium discs (respective p values of 0.024, 0.047 and 0.022). However at an inoculum 2×10^6^ CFU, the vancomycin-tethered discs were not significantly different from controls.

Experimental discs inoculated with increasing amounts of *S. aureus* were incubated overnight and the survival of metabolically active bacteria adhering to the disc surface was measured using resazurin reduction after thorough washing. Figure 2 shows resorufin fluorescence according to the initial bacterial inoculum used to seed the well. The distinction between measured fluorescence on vancomycin-bound discs and control titanium discs was large (>5-fold) for inocula of 2×10^3^, 2×10^4^ and 2×10^5^ CFU/disc (respective p values of 0.024, 0.047 and 0.022), However, at the highest inoculation of 2×10^6^ CFU/disc, fluorescence readings on the experimental discs approached levels of the control disks (1.5 fold ratio, p = 0.1), indicating that bacteria survived on the antibiotic tethered surface.

### The antimicrobial activity of soluble rifampicin was additive to vancomycin tethered to titanium discs

A checkerboard experiment performed on control discs showed that *S. aureus* ATCC 25923 had MICs of 0.5 mg/L to vancomycin and 0.015 mg/L to rifampicin using the standard 5×10^4^ CFU inoculum defined by CLSI. However, when applied in combination, sub-inhibitory concentrations of 0.25 mg/L of vancomycin and 0.008 mg/L of rifampicin were sufficient to inhibit growth, showing additivity of the two antibiotics. The possibility of synergism between vancomycin tethered to the titanium surface and soluble rifampicin was evaluated by determining the concentration of rifampicin capable of inhibiting the growth of *S. aureus* on experimental discs from an inoculum (5×10^6^ CFU) high enough to overload the intrinsic antibacterial properties of the vancomycin-tethered surface (Figure 2). A sub-inhibitory concentration of 0.008 mg/L of rifampicin was found to be sufficient to inhibit the growth on the experimental discs, whereas 0.015 mg/L rifampicin was required to inhibit the growth of 5×10^6^ CFU on control discs. Importantly, the MIC of rifampicin was not modified by the 100-fold increase of inoculum. These results show that like soluble vancomycin, titanium tethered vancomycin was additive with soluble rifampicin.

### Vancomycin tethered to titanium prevented the emergence of resistance to rifampicin

**Figure 3:**
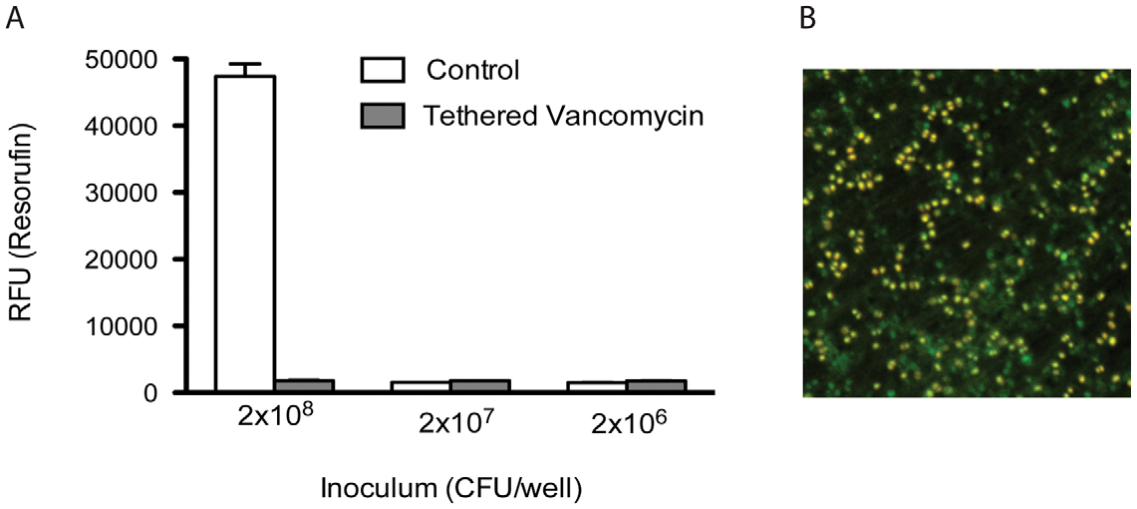
Vancomycin tethered titanium discs prevented the emergence of resistance to rifampicin. Vancomycin tethered and control discs were seeded with 2×10^6^, 2×10^7^ or 2×10^8^ CFU in presence of a suprainhibitory concentration of rifampicin (0.03 mg/L). Following centrifugation and incubation for 18 h, adhering bacteria were detected by resazurin assay and the discs were imaged by confocal laser scanning microscopy. Control discs inoculated with lower inocula were free of live cells, however, *S. aureus* grew in wells inoculated with 2×10^8^ CFU. **A** Resorufin assay: biofilm-forming bacteria reduce resazurin to resorufin on control discs inoculated with 2×10^8^ CFU/well, whereas vancomycin tethered discs prevented biofilm formation when inoculated with the same inoculum. **B** Confocal laser scanning microscopy shows that the surface of the control discs inoculated with 2×10^8^ CFU/well are colonized with a mixture of live (green, labeled by Syto9) and dead (yellow, co-labeled by Syto9 and propidium iodide) bacteria.

The ability of surface-tethered vancomycin to prevent the emergence of resistance to rifampicin was evaluated by measuring the metabolic activity of adherent bacteria from increasingly dense inoculations of *S. aureus* in the presence of 0.03 mg/L rifampicin (Figure 3A). Control discs inoculated with 2×10^6^ and 2×10^7^ CFU were sterile, as expected from their susceptibility to rifampicin. However, control discs inoculated with 2×10^8^ CFU were colonized with live bacteria that were determined to be rifampicin resistant with an MIC >4 mg/L. In contrast, vancomycin-tethered discs and the overlying medium were sterile when inoculated with 2×10^8^ CFU.

The surfaces of the discs inoculated with 2×10^8^ CFU were observed by confocal laser scanning microscopy (CLSM) after live/dead staining (Figure 3B). The vancomycin-tethered discs were free of adhering bacteria (data not shown) whereas the control discs were covered with a nascent biofilm populated with a mixed population of live as well as dead bacteria incapable of excluding propidium iodide. In the absence of the tethered vancomycin, the rifampicin-resistant mutants were capable of adhering to the surface and colonizing the medium and the disc surface.

## Discussion

Implant infection is a significant source of patient morbidity and is predicted to impact future healthcare costs [29]. Antibiotic resistance is persistent concern and revision surgery remains a highly invasive procedure. There will always be a risk of infection at the revision site since tissue spared to enable reconstruction may harbor an inoculum. As a result, the development of new approaches to treat implant related infections is of great interest. In this study, it was demonstrated that vancomycin tethered to titanium surfaces was effective for inhibiting the growth of *S. aureus* and preventing the development of resistance to rifampicin *in vitro*.

Rifampicin is the preferred drug against methicillin-resistant staphylococcal infection of implanted biomaterials, however, its major drawback is the high frequency of *rpoB* mutants (range from 10^−7^ to 10^−8^) exhibiting resistance following exposure [30], [31]. For the treatment of PJIs, rifampicin is often coadministered with another antibiotic agent due to the rapid emergence of bacterial resistance. Despite combination therapy, there are instances of selection of rifampicin resistant mutants due to the possible different pharmacokinetic properties of the combination agents [26], [30], [31]. Vancomycin is often co-administered with rifampicin, but the systemic delivery of vancomycin is not without difficulty. The pharmacokinetic/pharmacodynamic profile of vancomycin does not ensure serum levels capable of preventing resistance to rifampicin in the first days of therapy. As a result, it is common practice to delay the administration of rifampicin to ensure suitable levels of vancomycin are attained. This approach is common since administration of rifampicin in the absence of adequate levels of vancomycin could lead to a newly formed biofilm on the revised implant that is populated with rifampicin-resistant bacteria.

Vancomycin was selected for this study because it is used clinically to treat periprosthetic joint infections in combination with rifampicin [24]. Tethering vancomycin directly to the implant surface solves the problem of its poor tissue distribution relative to rifampicin, since it is present at an effective level *ab initio*. Vancomycin is suitable for tethering because of its stability, its amenability to covalent binding [15] and its mode of action. Because vancomycin acts on the outer bacterial wall it can remain tethered to the implant surface while interacting with biofilm forming micro-organisms.

The antibacterial activity of vancomycin tethered to titanium surfaces has been previously demonstrated [32], however the mechanism of action is not completely understood. Soluble vancomycin inhibits the cross-linking of *D*-Ala-*D*-Ala interpeptidic bridges in the peptidoglycan all around the bacterial cell wall whereas vancomycin tethered to titanium has limited cellular contact. The quantity of tethered vancomycin required to inhibit the activity of *S. aureus* is not understood either. In this study, the coverage of one vancomycin molecule per 3.8 nm^2^ is consistent with the reported aminopropylsilanation of 0.22 nmol/cm^2^ or 1 peptide per 0.8 nm^2^ by Spencer et al. [17]. Approximating the diameter of *S. aureus* as 1 µm, and that the titanium-tethered vancomycin has a limited reach of <5 nm from the metal surface, the bacterial wall area exposed to antibiotic is less than 1.5×10^4^ nm^2^, or 0.023% of the 6.5×10^7^ nm^2^ surface area (assuming a spherical cell and a compressible organic layer at the bacterium-implant interface). Furthermore, a coverage of one vancomycin molecule per 3.8 nm^2^ would allow <4000 molecules to interact with the cell wall of a given bacterium. The results of this study suggest that *in vitro*, 4000 vancomycin molecules or one molecule per 3.8 nm^2^ provide a sustained antibacterial effect.

This study determined that soluble rifampicin worked in conjunction with surface-tethered vancomycin *in vitro*. The results serve as an important proof of concept for more sophisticated experiments which could address pharmacokinetic questions. While promising, the effectiveness of this technique remains to be evaluated *in vivo* where stability of the tethered antibiotic may be an important issue. Silanization of the oxidized titanium surface with aminopropyltriethoxysilane is a versatile method for covalently attaching organic molecules to oxides. The resulting terminal amino group is amenable to further functionalization using standard solid-phase organic synthesis techniques. Although this silane-based chemistry is stable *in vitro* [33], concerns have been raised about its stability under physiological conditions [19]. *In vivo* studies of implants coated with silane-linked antibiotic [22] have demonstrated the potential for this chemistry to be sufficiently stable for use in medical devices. Furthermore, the general strategy employed in our current study could readily be adapted if necessary to potentially more robust phosphonate-based linking chemistries [19]. Another area for optimization would be to increase the access of the antibiotic to the bacterium. A flexible linker connecting vancomycin to the aminopropylsilyl anchor was used to allow the vancomycin molecule to adopt an effective position. Longer linkers may be advantageous by allowing a greater number antibiotic-bacteria interactions (*vide supra*), but may suffer from lower yields in the chemical coupling reactions. In this study, a previously validated aminoethoxyethoxyacetic acid dimer [14], which has a length <5 nm, was employed, but alternative linkers are under current investigation.

The use of implant-tethered vancomycin may provide a promising adjunct for the immediate postoperative use of rifampicin in the treatment of periprosthetic joint infections. The results of this study suggest that further validation of this approach is warranted. For example, the impact of the vancomycin tethering surface treatments on osseointegration should be determined, and evaluation in an animal model of PJI would provide additional, valuable information. While this approach has been developed to facilitate single stage revision for PJI, it could be applied to any titanium implant vulnerable to infection, from external pin fixation to dental implants or endovascular stents.
